# Phenotypical Characterization and Neurogenic Differentiation of Rabbit Adipose Tissue-Derived Mesenchymal Stem Cells

**DOI:** 10.3390/genes12030431

**Published:** 2021-03-17

**Authors:** Mária Tirpáková, Jaromír Vašíček, Andrea Svoradová, Andrej Baláži, Marián Tomka, Miroslav Bauer, Alexander Makarevich, Peter Chrenek

**Affiliations:** 1 AgroBioTech Research Center, Slovak University of Agriculture in Nitra, Tr. A. Hlinku 2, 949 76 Nitra, Slovakia; 2 Department of Biochemistry and Biotechnology, Faculty of Biotechnology and Food Sciences, Slovak University of Agriculture in Nitra, Tr. A. Hlinku 2, 949 76 Nitra, Slovakia; jaromir.vasicek@nppc.sk (J.V.); marian.tomka@uniag.sk (M.T.); 3 NPPC—Research Institute for Animal Production Nitra, Hlohovecká 2, 951 41 Lužianky, Slovakia; andrea.svoradova@nppc.sk (A.S.); andrej.balazi@nppc.sk (A.B.); miroslav.bauer@nppc.sk (M.B.); alexander.makarevic@nppc.sk (A.M.); 4 Department of Botany and Genetics, Faculty of Natural Sciences, Constantine the Philosopher University in Nitra, Nábrežie Mládeže 91, 949 74 Nitra, Slovakia

**Keywords:** rabbit, stem cells, adipose tissue, flow cytometry, digital droplet PCR, neural differentiation

## Abstract

Although the rabbit is a frequently used biological model, the phenotype of rabbit adipose-derived mesenchymal stem cells (rAT-MSCs) is not well characterized. One of the reasons is the absence of specific anti-rabbit antibodies. The study aimed to characterize rAT-MSCs using flow cytometry and PCR methods, especially digital droplet PCR, which confirmed the expression of selected markers at the mRNA level. A combination of these methods validated the expression of MSCs markers (CD29, CD44, CD73, CD90 and CD105). In addition, cells were also positive for CD49f, vimentin, desmin, α-SMA, ALDH and also for the pluripotent markers: NANOG, OCT4 and SOX2. Moreover, the present study proved the ability of rAT-MSCs to differentiate into a neurogenic lineage based on the confirmed expression of neuronal markers ENO2 and MAP2. Obtained results suggest that rAT-MSCs have, despite the slight differences in marker expression, the similar phenotype as human AT-MSCs and possess the neurodifferentiation ability. Accordingly, rAT-MSCs should be subjected to further studies with potential application in veterinary medicine but also, in case of their cryopreservation, as a source of genetic information of endangered species stored in the gene bank.

## 1. Introduction

In the last decades, interest in research on mesenchymal stem cells (MSCs) has increased due to their specific biological features. Owing to their ability to spread and differentiate, MSCs have found widespread use, not only in regenerative medicine, but also in various disease treatment therapies, veterinary medicine and drug development, as well as stem cell banking [1,2,3,4,5]. The greatest advances in stem cell-based therapy so far have been achieved with MSCs isolated from bone marrow. However, many studies have reported that MSCs can be obtained from different adult tissue sources, such as skin, skeletal muscle and adipose tissue [6,7,8].

Compared to bone marrow, adipose tissue possesses a multitude of advantages, not only in terms of better availability, but also easier and more affordable isolation. Moreover, in the favor of adipose tissue, the stem cell yield from the adipose tissue is predominantly higher compared to that from bone marrow [9,10]. The profile of MSC surface markers differs among species. Generally, according to the International Society for Cellular Therapy (ISCT) convention, MSCs from various sources, including adipose tissue-derived stem cells (AT-MSCs), are described in terms of phenotypes such as CD29^+^, CD44^+^, CD73^+^, CD90^+^, CD105^+^, and CD14^-^, CD34^-^, CD45^-^ [11,12,13,14]. In terms of characterization of AT-MSCs’ surface proteins, it is not appropriate to evaluate freshly isolated cells since no homogenous stem cell population can be obtained. It has been observed that AT-MSCs show altered expression of surface markers depending on the cell density and the number of passages [15,16]. Many studies are aimed at determination of AT-MSCs-specific surface markers using different techniques [8,17,18,19].

Stem cells derived from adipose tissue showed three lineage differentiation capacity in both in vitro and in vivo conditions. Despite their mesodermal origin, many studies have shown that they can likewise differentiate into the cells of ectodermal and endodermal origin. Generally, isolated AT-MSCs are induced to differentiation chemically using a culture medium supplemented with specific growth factors [10]. There are also novel approaches for the induction of differentiation employing the laser irradiation [10,20,21,22,23]. However, the changes in the morphology of AT-MSCs from fibroblast-like into neuron-like appearance have been associated not only with intended induction but also with cell shrinkage [24,25]. Therefore, morphological assessment alone cannot be an indicator of differentiation; thus, detection of the expression of neuronal markers is necessary. There is a wide range of neuronal markers dependent on the neuronal lineage being investigated, including glial fibrillary acidic protein (GFAP), microtubule-associated protein 2 (MAP2), nestin, neuron-specific enolase (ENO2) or β-III-tubulin [26,27].

Rabbit (*Oryctolagus cuniculus*) is commonly used as an experimental animal model for both human and veterinary medicine. Rabbit research is convenient due to animal body size, their ease to breed and low cost of their feeding and management [28]. Although they are comparatively larger than mice, rabbits have a shorter gestation period, which permits the use of a high number of animals and they also are phylogenetically closer to primates than rodents [29,30]. Thus, the objective of this study was to establish a comprehensive characterization of rabbit adipose tissue-derived stem cells, since there are only a few studies aimed at this topic. Hence, this study is mainly focused on the phenotyping of rabbit adipose tissue-derived stem cells by flow cytometry, reverse transcription-polymerase chain reaction (RT-PCR) and droplet digital PCR (ddPCR). Moreover, we examined the neurogenic differentiation potential of these cells in comparison to rabbit mesenchymal stem cells from other sources.

## 2. Materials and Methods

### 2.1. Ethical Standards

Authors proclaim that all procedures conducted in this work abide by the ethical standards of the relevant national and institutional guidelines on the care and use of laboratory animals. The treatment of the animals was approved by the Ministry of Agriculture and Rural Development of the Slovak Republic no. SK U 18016 in accordance with the ethical guidelines presented in Slovak Animal Protection Regulation, RD 377/12, which conforms to European Union Regulation 2010/63.

### 2.2. Animals

Clinically healthy rabbit females of the New Zealand White (NZW) line were used in the study. Rabbits were reared in a partially air-conditioned hall of rabbit farm of the Institute of Small Farm Animals at the NPPC-Research Institute for Animal Production Nitra, Slovakia. Housing conditions and preparation of females (hormonal stimulation and artificial insemination) were performed as described in a previous study [31]. The rabbits were fed ad libitum with a commercial feed mixture (KV, TEKRO Nitra Ltd., Nitra, Slovakia) and the water was provided ad libitum using water feeders. 

### 2.3. Collection and Processing of the Biological Material

Rabbit females were humanely sacrificed at Day 23 of gestation and amniotic fluid was recovered from a withdrawn uterus and, subsequently, rabbit femurs were dissected. Processing of amniotic fluid and bone marrow was described in our previous studies [31,32,33]. Concurrently subcutaneous fat was harvested. The collected fat samples were washed with phosphate-buffered saline (PBS) (without Ca^2+^ and Mg^2+^ ions; Biowest, Riverside, MO, USA) containing 5% penicillin/streptomycin antibiotics (Thermo Fisher Scientific, Waltham, MA, USA). Following washing, the debris (blood vessels, connective tissue, muscle tissue, etc.) was removed using scissors and tweezers. Adipose tissue was cut into small pieces and rewashed with a PBS containing antibiotics. Samples were centrifuged at 500× *g* for 5 min.

### 2.4. Isolation and Culture of Rabbit Stem Cells

Adipose tissue samples were incubated at 37 °C for about 2 h with collagenase type I (Sigma Aldrich, Gillingham, UK) at a concentration of 0.2%. The enzymatic solution was neutralized with a culture medium and filtered through a 100 µm filter to remove the undigested tissue. After filtration, the samples were centrifuged at 1200× *g* for 10 min. Following centrifugation, cell pellets were resuspended in Gibco^TM^ αMEM culture medium (Thermo Fisher Scientific) supplemented with 20% fetal bovine serum (Sigma Aldrich, Gillingham, UK) and 1% penicillin/streptomycin antibiotics (Thermo Fisher Scientific). The medium was changed every 3 days to remove non-adherent cells. Stem cells isolated from the adipose tissue (AT-MSCs) reached 90% confluency in about 6–7 days after isolation. Cells were cultured until passage 3 (P3), as previously described [34]. Isolation and culture of stem cells from the amniotic fluid (AF-MSCs) and the bone marrow (BM-MSCs) were described in previous studies [31,32,33]. Briefly, amniotic fluid was diluted (1:1) with a culture medium; EBM-2 basal medium (Lonza, Walkersville, MD, USA) supplemented with 20% fetal bovine serum (Sigma Aldrich), EGM-2 SingleQuots™ Kit (Lonza), and 1% penicillin/streptomycin. Femoral bone heads were removed under sterile conditions and bone marrow was flushed using PBS (without Ca^2+^ and Mg^2+^ ions). After filtration the cell suspension was layered on a Biocoll (Biochrom, Berlin, Germany) and separated using density gradient centrifugation at 867× *g* and 20 °C for 20 min. Density of cell seeding was as follows: 1.2 × 10^4^ cells/cm^2^ for amniotic fluid and adipose tissue and 1.2–1.5 × 10^6^ cells/cm^2^ for the bone marrow. All types of rabbit stem cells were maintained under the same conditions at 37 °C and a 5% CO_2_ in the atmosphere.

### 2.5. Culture of Human Adipose-Derived Stem Cells

Commercially available human AT-MSCs (hAT-MSCs; C-12977, PromoCell, Heidelberg, Germany) were obtained at passage 2. Cells were cultured in Gibco^TM^ αMEM culture medium (Thermo Fisher Scientific) supplemented with 20% of fetal bovine serum (Sigma Aldrich) and 1% of antibiotic/antimycotic solution (Biowest). Cells were seeded on 75 cm^2^ culture flasks at a density of 1.2 × 10^4^ cells/cm^2^ and maintained under standard conditions at 37 °C and a 5% CO_2_ in the atmosphere.

### 2.6. Population Doubling Time

In order to determine the population doubling time (PDT), cells were counted at every passage (P1–P3) and culture time was recorded. Cells were dissociated and concentration was counted as we described in our previous study [34]. Population doubling time was counted for each passage by the growth curve using the doubling time calculator available at http://www.doubling-time.com/compute.php (5 December 2020). 

### 2.7. Detection of Surface and Intracellular Markers Using Flow Cytometry

To confirm the origin of rabbit BM-MSCs, AF-MSCs and AT-MSCs, the detection of the cell surface and intracellular markers was performed by an antibody immunofluorescent staining, as described in our previous studies [31,32]. The cells were double-stained using a rat anti-mouse IgG1-PE fluorochrome-conjugated secondary antibody (clone X-56; Miltenyi Biotec, Bergisch Gladbach, Germany) or goat anti-mouse IgG-FITC polyclonal antibody (STAR117F, Bio-Rad, Hercules, CA, USA). A complete list of primary antibodies with an indication of their reported reactivities, used in this study, is shown in Table 1. To exclude the dead cells from the analysis, samples were co-stained with dead cell marker such as 7-AAD (eBioscience, Wien, Austria). Cells were analyzed using a FACS Calibur ™ device (BD Biosciences, San Jose, CA, USA) and Cell Quest Pro ™ software (BD Biosciences). At least 50,000 events were analyzed for each sample. Unstained FMO (fluorescence minus one) samples were used as control samples in order to gated the positive cells according to the increased fluorescent intensity.

ALDH activity was assessed using the ALDEFLUOR™ kit (STEMCELL Technologies, Vancouver, BC, Canada) and evaluated using flow cytometry. Briefly, cells were incubated with an Aldefluor substrate (15 min; 37 °C) with or without the ALDH inhibitor diethylamino-benzaldehyde (DEAB) in accordance with the manufacturer’s guidelines. Stained cells were analyzed by a flow cytometer (FACSCalibur, BD Biosciences). At least 25,000 cells were analyzed in each sample. 

### 2.8. Detection of Surface and Intracellular Markers Using Confocal Microscopy

For the visualization of the selected rMSCs markers an immunofluorescence assay was performed. Briefly, approximately 3 × 10^4^ cells from the passage 2 (P2) were resuspended in culture medium and allowed to adhere to a microscopic slide placed into a 4-well plate (NUNC) at 37 °C in a 5% CO2 humidified atmosphere until reaching 80% confluency. For surface markers CD90, CD105 and pluripotent markers SOX2, NANOG, OCT4, the cells were pre-fixed using an IC Fixation Buffer (Thermo Fisher Scientific). In addition, nuclear markers SOX2, NANOG and OCT4 required permeabilization of cells with 0.1% Triton X-100. Pre-fixation and permeabilization with acetone:methanol (1:1) mixture was applied for intracellular cytoplasmic markers (vimentin, desmin, α-SMA) and ALPL. Thereafter, the cells were gently washed with PBS and incubated with primary antibodies overnight. Cells stained for CD29, CD49f and CD73 were incubated for 20 min, washed and post-fixed with an IC Fixation Buffer. Afterward, cells were washed with PBS and incubated with an adequate secondary antibody (Table 2). Following the final cell wash with PBS, 4 μL of Vectashield anti-fade mounting medium containing DAPI nuclear stain (Vector Laboratories, Burlingame, CA, USA) were pipetted on a microscope slide. Lastly, a coverslip with adhered cells was carefully placed on a microscope slide with the cell-coated side down. Stained cells were evaluated using an LSM 700 laser scanning confocal microscope (Carl Zeiss Slovakia, Bratislava, Slovak Republic).

### 2.9. RT-PCR

RT-PCR analyses were carried out to detect mRNA expression of specific cell surface markers. Total RNA from 3–5 × 10^6^ rabbit stem cells was isolated using TRI Reagent^®^ RT (Molecular Research Center, Cincinnati, OH, USA) according to the manufacturer’s protocol. The purity of extracted RNA was determined by UV spectrophotometry at 260/280 nm ratio and the integrity of RNA was checked by electrophoresis in 1% agarose gel. In order to destroy contaminating DNA, before reverse transcription RNA, samples were treated with the dsDNase (Thermo Fisher Scientific). The first-strand cDNA was synthesized using Maxima H Minus First Strand cDNA Synthesis Kit (Thermo Fisher Scientific) with 1.5 µg of total RNA from each sample, oligo (dT)_18_ and random hexamer primers in a total volume of 20 µL. 

The reaction was performed at 25 °C for 10 min, then at 55 °C for 30 min, and terminated at 85 °C for 5 min. A PCR was performed in 20 µL reactions containing 1 µL cDNA, 4 µL of 5× MyTaq reaction buffer, 1U of MyTaq HS DNA polymerase (Bioline, Memphis, TN, USA), and 5 pmol of each primer for tested markers (Table 3) using C1000 Thermal Cycler (Bio-Rad). Rabbit β-2-microglobulin (B2M) was applied as a reference gene, and the amplification protocol for all genes was as follows: an initial denaturation and activation of Taq DNA polymerase at 95 °C for 2 min, followed by 35 cycles of denaturation at 95 °C for 15 s, annealing at 60 °C for 15 s and polymerization at 72 °C for 15 s. The final polymerization step was extended to 5 min at 72 °C. PCR products were electrophoretically separated in 2% agarose gel in TAE buffer.

### 2.10. Digital Droplet PCR

In order to quantify the expression of chosen markers at the mRNA level, we used a novel method of digital droplet PCR (ddPCR). The reaction mixture was prepared according to the manufacturer’s protocol and contained 10 μL of QX200™ ddPCR™ EvaGreenSupermix (Bio-Rad), 1 μL of cDNA, 0.5 μL of primers and was filled to the final volume of 20 μL with ultrapure water. Thereafter, prepared suspension was divided into individual tubes. To form droplets, 20 µL of the reaction mixture were mixed with 70 µL of oil, and samples were afterward pipetted onto a droplet generation cartridge DG8^TM^ plate for the QX200^TM^ Droplet Generation Oil for EvaGreen system (Bio-Rad). The loaded cartridge was covered with a DG8 Gasket and placed into the QX200 Droplet Generator. Once the droplet generation is completed, droplets were pipetted onto a PCR 96-well plate (Bio-Rad) and sealed prior to the PCR reaction. The sealed plate was placed into a T100 thermal cycler (Bio-Rad), where the PCR reaction took place under the following conditions: initial denaturation and activation of hot-start DNA polymerase at 95 °C for 2 min followed by 40 cycles of denaturation at 95 °C for 15 s, annealing at 60 °C for 15 s and extension at 72 °C for 15 s. When PCR amplification is complete, droplets were read using the QX200 Droplet Reader (Bio-Rad), where the individual droplets were evaluated separately based on the fluorescence signal. The results were evaluated using Quanta Soft version 1.7.4.0917 (Bio-Rad). To achieve the most accurate results, only samples containing at least 12,000 droplets were used for quantification. The results were expressed as a ratio of the number of positive droplets to the total number of droplets in the sample. 

### 2.11. Neurogenic Differentiation

To confirm the potential of neurogenic differentiation of rabbit stem cells, the cells at P2 were seeded with density of 1.0 × 10^4^ cells per cm^2^ and were cultured in a standard culture medium supplemented with 20% of FBS and 1% antibiotics. After reaching about 80% confluency, cells were detached as described above and reseeded on 75 cm^2^ tissue culture flasks with a density of 1.0 × 10^4^ cells per cm^2^. After 48 h, cells became sub-confluent (about 80%), culture medium was discarded, cells were washed with PBS and the medium was replaced with a mesenchymal stem cell neurogenic differentiation medium (PromoCell, Heidelberg, Germany). Differentiation of rabbit stem cells into neurogenic lineage was performed according to the manufacturer’s instructions under standard growth condition (37 °C; 5% CO_2_). The medium was changed after 48 h. After 3 days of induction, cells were detached and used for further analyses. In addition, the typical three-lineage differentiation potential of these cells was analyzed (Appendix A).

### 2.12. RT-qPCR

Total RNA isolation from rabbit stem cells (rBM-MSCs, rAF-MSCs, rAT-MSCs) and cDNA synthesis were done as described above. A PCR was performed in 20 μL parallel reactions containing 1 μL of cDNA, 10 μL of DyNAmo Flash SYBR Green PCR mix (Thermo Fisher Scientific), and 5 pmol of each primer for MAP2, ENO2, and β-2-microglobulin (B2M), as a reference gene (Table 4) in Rotor-Gene 6000 device (Corbett Research, Sydney, Australia). The amplification protocol was the following: an initial denaturation and activation of Taq DNA polymerase at 95°C for 7 min followed by 40 cycles of 95 °C for 10 s, 60 °C for 10 s and 72 °C for 10 s. To check the specificity of PCR products, a melting curve analysis within a temperature range of 72–95 °C as well as electrophoresis in 2% agarose gel were performed. The standard curves were generated for all genes using a serial dilution of template cDNA. Relative quantification of MAP2 and ENO2 expression to reference B2M gene was calculated using the threshold (C_T_) values and PCR reaction efficiencies according to Pfaffl [36]. In respect to the hAT-MSCs, the same protocol was applied using primers specified in Table 4. 

### 2.13. Fluorescent Assessment of Neurodifferentiation

Successful neurodifferentiation was confirmed based on the chosen markers microtubule-associated protein 2 (MAP2) and neuron-specific enolase (ENO2) using confocal microscopy. Cell culture was assessed after three days of induction of differentiation into neurogenic lineage: starting since overnight incubation with a MAP2 primary antibody (Clone BB7, Creative Diagnostics, Shirley, NY, USA) or ENO2 (Clone NSE47, Enzo Life Sciences, Farmingdale, NY, USA), followed by washing and consequent incubation with a goat anti-mouse IgG-FITC secondary antibody (STAR117F, Bio-Rad).

### 2.14. Statistical Analysis

The results were evaluated with the descriptive statistics or a Student’s t-test (for RT-qPCR) using the SigmaPlot software (Systat Software Inc., Erkrath, Germany). The values are expressed as the means ± SD.

## 3. Results

### 3.1. Morphology and Proliferation of Rabbit AT-MSCs

**Figure 1:**
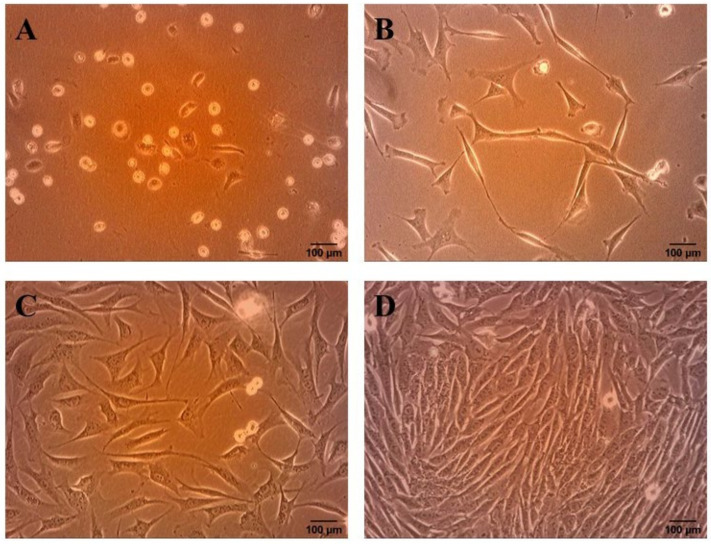
Morphological changes in rAT-MSCs during the culture. (**A**): Cells started to adhere to culture flasks 24 h after seeding; (**B**): the cells began to cluster into small colonies 72 h in culture; (**C**): the cells reached confluency approximately 50–60% on the 5th day; (**D**): cell culture consisted of a homogenous monolayer of fibroblast-like cells on the 7th day after isolation (scale bar = 100 µm).

Immediately after seeding, the cells with round shape were observed. After 24 h of plating, cells started to adhere to tissue culture flasks and their morphology changed into spindle-shaped (Figure 1A). The medium was replaced every 2 days to remove non-adherent cells. On the approximately third day, cells began to cluster into small colonies, proliferated rapidly and reached about 50–60% confluency (Figure 1B,C). After 6–7 days the cells reached 90% confluency and the culture consisted of a homogenous monolayer of fibroblast-like cells (Figure 1D). PDT was calculated basing on the cell number counted after detachment and the culture period. The average PDT for rAT-MSCs in our study was 37.45 ± 1.32 h. 

### 3.2. Detection of the Expression of Surface and Intracellular Markers Using Flow Cytometry

Analysis of the phenotype of rMSCs showed high positivity of CD29, CD44, CD49f as well as intracellular markers—vimentin, desmin and α-smooth muscle actin (α-SMA). The expression of CD73, CD90, and CD105 markers was not of the expected percentage, therefore, these antibodies were substituted by available alternatives of higher affinity. New anti-human antibodies for detection of CD73, CD90 and CD105 determined higher expression, especially for the surface marker CD90. High expression (over 90%) of CD73, CD90 and CD105 was confirmed in the case of hAT-MSCs. The activity of aldehyde dehydrogenase (ALDH) was highly positive (more than 70%) only in rAT-MSCs, rBM-MSCs and also in hAT-MSCs. Markers of hematopoietic lineage (CD34 and CD45), used as a negative control, were not expressed by any type of rMSCs. The expression was represented as the mean (%) ± SD, separately for each marker (Table 5). 

### 3.3. Detection of Surface and Intracellular Markers Using Confocal Microscopy

**Figure 2:**
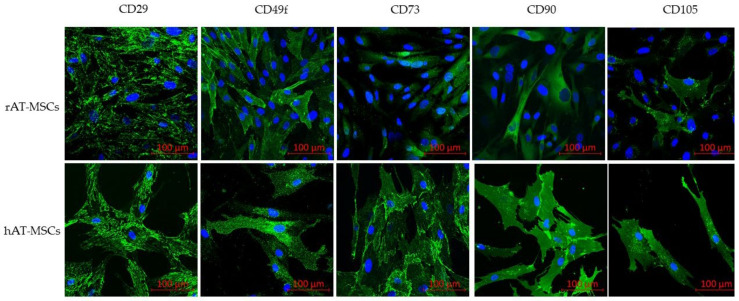
Immunofluorescence of selected surface markers of rabbit (rAT-MSCs) and human (hAT-MSCs) samples (scale bar = 100 µm).

**Figure 3:**
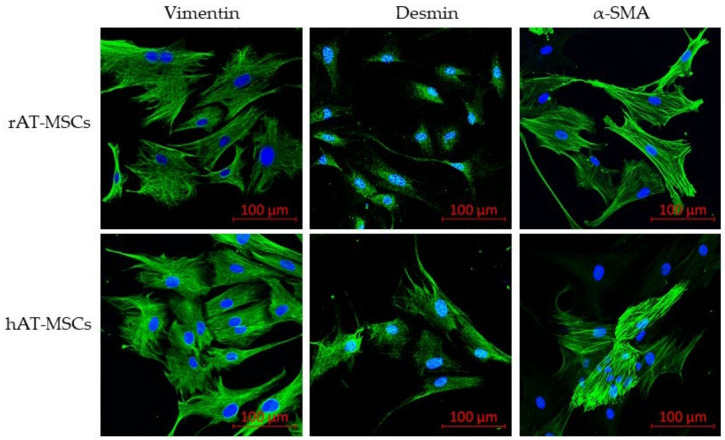
Immunofluorescence of selected intracellular markers of rabbit (rAT-MSCs) and human (hAT-MSCs) samples; α-SMA—α smooth muscle actin; (scale bar = 100 µm).

**Figure 4:**
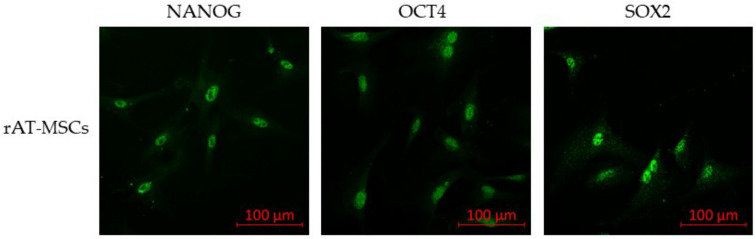
Immunofluorescence of selected pluripotent markers of rabbit (rAT-MSCs) samples; SOX2—sex determining region Y—box 2; OCT4—octamer-binding transcription factor 4; (scale bar = 100 µm).

To confirm the phenotype, confocal microscopy proved the expression of all tested surface markers (Figure 2) and intracellular markers (vimentin, desmin and α-SMA; Figure 3) in both rabbit and human AT-MSCs. Moreover, immunofluorescent staining of rAT-MSCs showed positive expression of the selected pluripotent markers NANOG, OCT4 and SOX2 (Figure 4). 

### 3.4. RT-PCR

**Figure 5:**
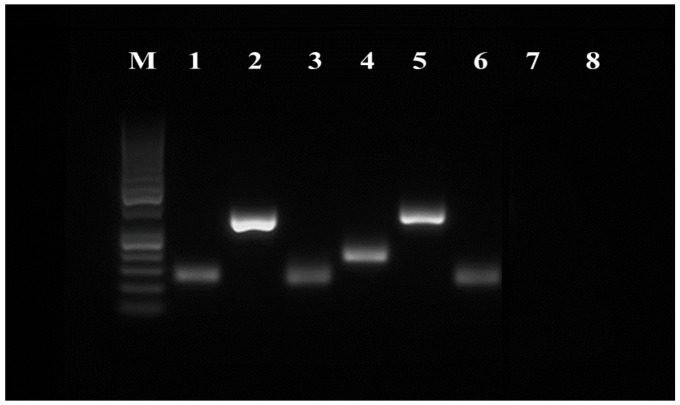
RT-PCR analysis of the expression of rAT-MSCs surface markers. Lane M—50 bp DNA ladder (Thermo Fisher Scientific, USA); lane 1—B2M (control); lane 2—CD29; lane 3—CD44; lane 4—CD73; lane 5—CD90; lane 6—CD105; lane 7—CD34; lane 8—CD45.

**Figure 6:**
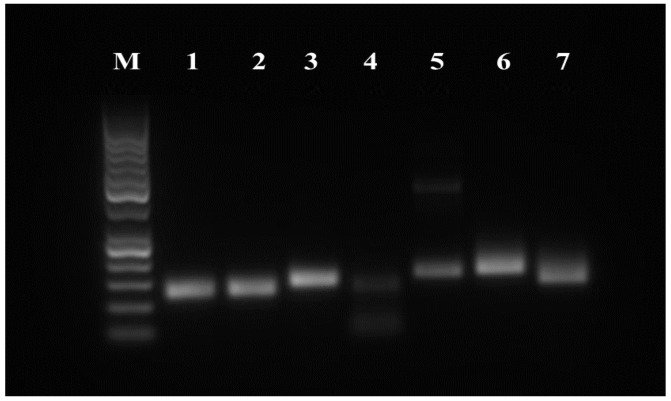
RT-PCR analysis of the expression of rAT-MSCs pluripotency markers. Lane M—50 bp DNA ladder (Thermo Fisher Scientific, USA); lane 1—B2M (control); lane 2—ST3GAL2; lane 3—ALPL; lane 4—NANOG; lane 5—OCT4; lane 6—SOX2; lane 7—ALDH.

The expression of surface and pluripotency markers was assessed at the mRNA level using the RT-PCR method. The following cell surface markers were examined: CD29, CD44, CD73, CD90, CD105, CD146, CD166, CD34 and CD45. Cell pluripotency markers (NANOG, OCT4 and SOX2) and other stem cell-specific markers (ST3GAL2 and ALDH) were also monitored. The rabbit β-2 microglobulin (B2M) was used as a reference gene. The results of RT-PCR analyses confirm that rMSCs express all CD surface markers characteristic for MSCs (CD29, CD44, CD73, CD90 and CD105). The markers of the hematopoietic line (CD34 and CD45) were not expressed in tested samples (Figure 5). Rabbit AT-MSCs also expressed pluripotent markers OCT4 and SOX2, and a weak signal was recorded also for NANOG. The presence of ST3GAL2 and ALDH markers was also verified (Figure 6).

### 3.5. Droplet Digital PCR

In each sample, an average of 12,000 droplets were evaluated. The results of the analyses were expressed as the average percentage of positive droplets ± SD. Results are summarized in Table 6. Results obtained from ddPCR indicate high expression of all selected markers. Marker of hematopoietic lineage (CD45), used as a negative control, was not expressed in rMSCs. 

### 3.6. Neurodifferentiation of Rabbit Stem Cells

#### 3.6.1. RT-qPCR

**Figure 7:**
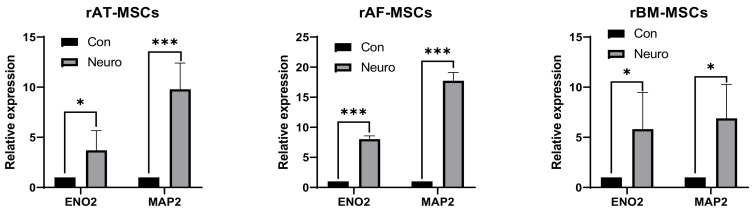
Results of neural marker gene expression using RT-qPCR. * *p* < 0.05; *** *p* < 0.001; Con—control (non-induced sample), Neuro—neurodifferentiated sample; rAT-MSCs—rabbit adipose tissue-derived mesenchymal stem cells; rAF-MSCs—rabbit amniotic fluid mesenchymal stem cells; rBM-MSCs—rabbit bone marrow mesenchymal stem cells; ENO2—neuron-specific enolase; MAP2—microtubule-associated protein 2.

Analysis of neural gene expression using quantitative real-time PCR was performed to evaluate the differentiation of rMSCs induced with a specific neuronal differentiation culture medium. The results displayed a significantly higher level of gene expression in differentiated cells for both markers ENO2 and MAP2 already on the 3rd day of induction (Figure 7).

**Figure 8:**
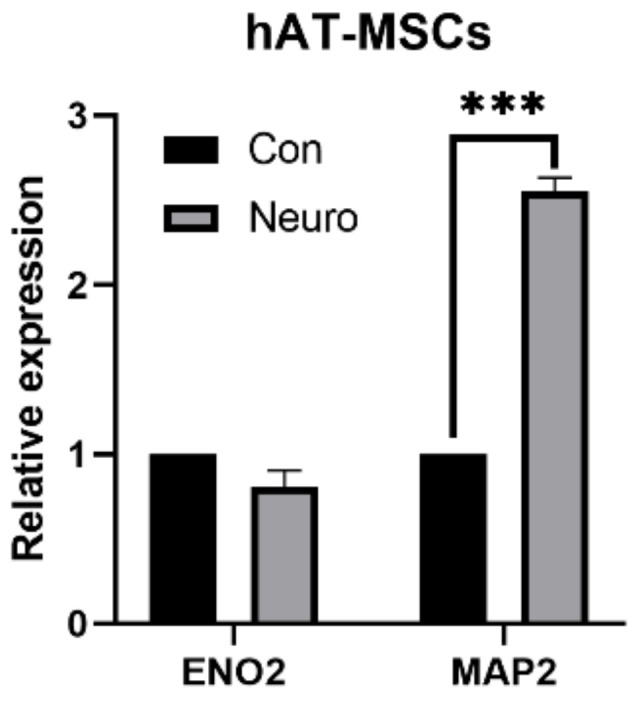
Expression of neural markers of human adipose tissue-derived mesenchymal stem cells (hAT-MSCs); *** *p* < 0.001; Con—control (non-induced sample), Neuro—neurodifferentiated sample; ENO2—neuron-specific enolase; MAP2—microtubule-associated protein 2.

In the case of hAT-MSCs, enhanced expression of ENO2 was not noticed in the group of differentiated cells. In contrast, the expression of the MAP2 marker was significantly increased in the group of differentiated cells (Figure 8).

#### 3.6.2. Confocal Microscopy

**Figure 9:**
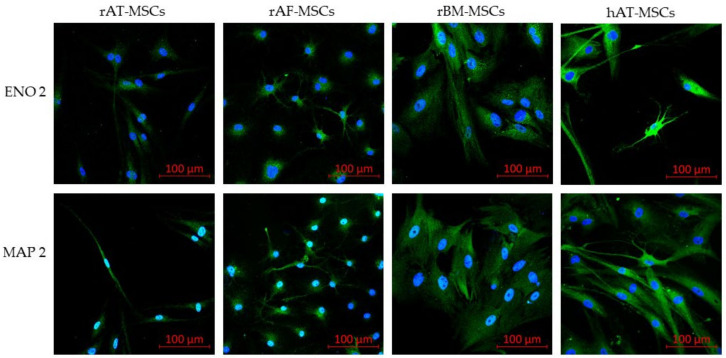
Confocal microscopy of specific neural markers. Neural markers ENO2 and MAP2 were highly expressed in all differentiated rabbit mesenchymal stem cells (rMSCs) as well as in human adipose tissue-derived mesenchymal stem cells (hAT-MSCs); rAT-MSCs—rabbit adipose tissue-derived mesenchymal stem cells; rAF-MSCs—rabbit amniotic fluid mesenchymal stem cells; rBM-MSCs—rabbit bone marrow mesenchymal stem cells; ENO2—neuron-specific enolase; MAP2—microtubule-associated protein 2; (Scale bars = 100 µm).

The expression of specific proteins of neuronal cells was examined by confocal microscopy. Immunofluorescent staining of differentiated cells proved the presence of specific neuronal markers, including neuron-specific enolase (ENO2) and microtubule-associated protein 2 (MAP2), in rMSCs and in hAT-MSCs (Figure 9).

## 4. Discussion

Adipose tissue has come to the forefront of many studies, mainly due to its availability, easier isolation and higher cell yields, compared to the bone marrow. From a morphological point of view, these cells show a fibroblast-like shape, which changes during the culture from round to spindle-shaped. Similar cell morphology has been confirmed by many studies not only in rabbits [38,39] but also in other animal species, such as dogs [40], pigs [41], horse [42,43] and humans [44,45]. To monitor the rate of proliferation, we evaluated the doubling time of the cell population (PDT). We calculated the doubling time separately for each passage since P1 to P3. The mean PDT values for the individual passages were 38.97 ± 14.05 h (P1), 36.26 ± 8.58 h (P2) and 37.11 ± 15.10 h (P3). From the results of the study [33], which reported PDT of rBM-MSCs for approximately 5 days, we concluded that rAT-MSCs proliferate significantly faster. Longer doubling time of the population was also reported for rAF-MSCs (61.5 ± 16.5 h) [32]. Similar results for hAT-MSCs are described [46], and the results of their study contradict many claims about the effect of age on cell proliferative activity. When comparing the proliferation of the adipose tissue and the bone marrow stem cells, AT-MSCs showed a higher rate of expansion compared to BM-MSCs, which was confirmed in humans [47], but also in rats [48] and guinea pigs [49]. When comparing the PDT of human stem cells from different sources (placenta, bone marrow, umbilical cord, adipose tissue and amniotic fluid), the PDT of adipose stem cells was significantly lower compared to other sources, suggesting that these cells show the best in vitro proliferation activity [40].

The phenotype of human AT-MSCs is thoroughly characterized in various studies [13,47]. In general, these cells are defined as CD29^+^, CD44^+^, CD73^+^, CD90^+^, CD105^+^, CD34^−^ and CD45^−^. A similar phenotype, even though with small differences, was confirmed also for rabbit AT-MSCs [39,50]. The positivity for CD49f marker (integrin α6), which is associated with cell pluripotency, is described by the present study in accordance with previous reports of [51,52,53], who claimed the expression of CD49f in MSCs from various sources. The present study contains the results confirming the expression of intracellular markers vimentin, desmin, αSMA, and the activity of aldehyde dehydrogenase (ALDH). The level of ALDH is currently used as a selection marker of the stem cells due to its relation to self-renewal and differentiation abilities [54,55]. The phenotypic profile was confirmed by flow cytometry and PCR methods on the mRNA level. The positive expression of surface and intracellular markers was confirmed by confocal microscopy in both rAT-MSCs and hAT-MSCs. Studies comparing the phenotype of human and rabbit AT-MSCs [39,50,56] point out discrepancies in the expression of CD73, CD90 and CD105. Different clones of antibodies for these markers were tested on various rMSCs to distinguish the most appropriate antibodies for rabbit species. Selected markers were found highly positive using the digital droplet PCR technique. Thus, in the case of the unavailability of specific rabbit-antibodies, it is inevitable to quantify the expression applying other methods. According to the described results and previously published data, the use of other methods, such as RT-PCR or ddPCR, ought to be conducted in addition to flow cytometry for the purpose of appropriate phenotypic analysis of rabbit MSCs. 

A difference between human BM-MSC and AT-MSC was observed in CD34 expression. While AT-MSCs weakly express this marker, in BM-MSC culture at the first passages, such expression was not confirmed [56,57]. On the other hand, an intracellular expression of CD34 was observed in the latter passages of human AT-MSCs [58]. Here, we did not observe any surface expression of CD34 in rAT-MSCs, although the intracellular expression of this marker was not analyzed. However, as we have already reported in our previous study [59], there is a lack of truly specific anti-rabbit CD34 antibodies that might be used for the immunological diagnostic methods. Nevertheless, also RT-PCR method did not reveal any CD34 expression, even if it would be expressed intracellularly. Moreover, the specificity of the PCR primers used to detect rabbit CD34 expression was validated in our previous studies [33,59]. Some studies also point to differences in the expression of intracellular markers [11,60]. The results indicate a difference in the expression of desmin, which was better expressed in BM-MSCs. Likewise, while desmin expression was positive in rabbit cells, human bone marrow stem cells did not express this marker. On the contrary, based on the results of our previous and present studies, we can state that this marker was more expressed in rAT-MSCs, rather than in rBM-MSCs, while we did not test this marker in hAT-MSCs. Despite the differences in isolation and culture, the immunophenotypic profile of rabbit AT-MSCs is relatively similar to the stem cells derived from the bone marrow and amniotic fluid, what was eventually confirmed by our previous [32,33] and the present studies. These results suggest that, although rabbit and human mesenchymal stem cells have similar differentiation potential, the expression of surface and intracellular markers differs among species. The expression of both surface and intracellular markers might be affected by in vitro culture and increasing passage number. 

The expression of pluripotent markers in MSCs is controversial. While some authors [61,62] pointed out that AT-MSCs express embryonic stem cell genes, including OCT4, the findings of other authors did not confirm OCT4 expression in human [63] and murine [64] AT-MSCs. In our previous studies [31,33], we observed differences in the expression of pluripotent markers with respect to the source of stem cells. While rAF-MSCs expressed all selected pluripotency markers (NANOG, OCT4 and SOX2), rBM-MSCs expressed only the SOX2 marker. Compared to the results obtained in the present study, we assume that rAT-MSCs have better differentiation potential than rBM-MSCs, as we confirmed the expression of all mentioned markers of pluripotency by RT-PCR and confocal microscopy in those cells.

**Figure A1:**
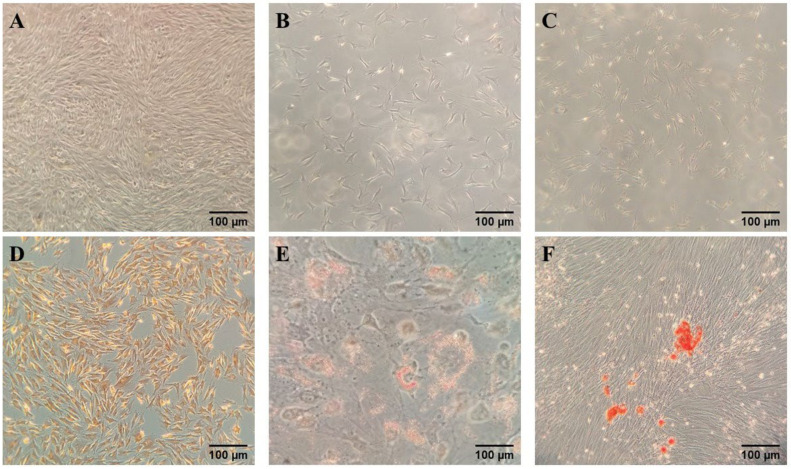
Histological staining of rAT-MSCs. (**A**–**C**)—Non-differenced cells remained unstained; (**D**)—deposits of proteoglycan in the differentiation-induced sample are stained with Safranin-O; (**E**)—lipid drops are stained red by Oil-Red-O; (**F**)—red dye Alizarin-Red-S identifies accumulation of calcium aggregates (Scale bars = 100 µm).

In general, hAT-MSCs are capable of differentiating into three cell lines: chondrocytes, osteocytes and adipocytes. The differentiation potential of stem cells is assessed in vitro using standard culture conditions in specific differentiation media. Commercial kits containing special media supplements, histological staining solutions or antibody panels are currently being designed to evaluate the differentiation of various cell lines. For more accurate analysis of differentiation, quantitative evaluation using lineage-specific gene markers is recommended [65]. In the present work, we differentiated rAT-MSCs into three baselines using commercially available differentiation kits. Based on the results of histological staining, we confirmed the successful differentiation of cells into osteogenic, adipogenic and chondrogenic lines (Figure A1; Appendix A). Our results are consistent with many studies on the differentiation potential of AT-MSCs, not only in rabbits but also in rats, guinea pigs, horses and also in humans [48,49,63,64,65]. In our previous studies, we also confirmed this differentiation potential on rBM-MSCs and rAF-MSCs [32,33]. 

In addition to basic three lineage differentiation, AT-MSCs have been shown to have neurogenic differentiation potential [66] and can also differentiate into cardiomyocytes and myocytes [67], endothelial cells [68] or hepatocytes [69]. The capability of AT-MSCs to differentiate into neuro-lineage possesses an outstanding potential for treating various neurological disorders. According to the literature, the changes in the morphology of AT-MSCs from fibroblast-like into neuron-like appearance recognized during short chemical induction, may be caused as a result of the cell shrinkage but not neural differentiation [24,25]. Similarly, histological staining of differentiated cells may not be conclusive evidence of neurodifferentiation. Hereby, morphological changes and histological staining alone should not be considered as successful differentiation, but the evaluation should be complemented by the detection of the expression of specific markers. There is a wide range of neuronal markers dependent on the neuronal lineage being investigated, including glial fibrillary acidic protein (GFAP), microtubule-associated protein 2 (MAP2), nestin, neuron-specific enolase (ENO2) or β-III-tubulin [26,27]. The expression of specific neuronal markers (ENO2, MAP2) was confirmed in the present work in all types of rabbit MSCs (AT-MSCs, BM-MSCs and AF-MSCs) as well as in hAT-MSCs, similarly as in other studies on human MSCs from different sources [70,71]. Based on our results, which correspond to the previously reported findings [72,73,74], we can state that stem cells isolated from the adipose tissue show several advantages compared to the bone marrow. Therefore, further examination of AT-MSCs is necessary to increase the quality and safety of clinical use in both human and veterinary medicine. The brief characteristics of rabbit and human mesenchymal stem cells isolated from different biological sources are listed in Table 7.

## 5. Conclusions

In summary, the present study was focused on properties of rabbit AT-MSCs. Obtained results suggest high similarity between rabbit AT-MSCs and human AT-MSCs. Moreover, it suggests the need for the assessment of marker expression at the mRNA level. The combination of immunostaining and PCR methods resulted in confirmation of positive expression of surface and intracellular markers (CD29, CD44, CD49f, CD73, CD90, CD105, vimentin, desmin, α-SMA and ALDH). In addition, the expression of pluripotent markers (NANOG, OCT4 and SOX2) was confirmed. Based on these findings we can point out that successful neurodifferentiation was induced in rMSCs culture, which was proved by the presence of specific neuronal markers (ENO2 and MAP2). In conclusion, further analyses of rAT-MSCs are required in order to provide additional characterization of these cells intended for both clinical application and cryopreservation. AT-MSCs, cryostored in a gene bank, may serve as a valuable genetic source of breeds threatened with extinction.

## Figures and Tables

**Table 1:** List of primary antibodies used for flow cytometry.

Marker	Host	Reactivity	Clone	Conjugate	Company
CD29	mouse IgG1	rabbit	P4G11	FITC	Merck
CD34	mouse IgG1	human	QBEnd-10	FITC	Thermo Fisher Scientific
CD44	mouse IgG1	rabbit	W4/86	-	Bio-Rad
CD45	mouse IgG1	rabbit	L12/201	-	Bio-Rad
CD49f	rat IgG2a	rabbit	GoH3	AF647	Biolegend
CD73	rat IgG1	mouse	TY/11.8	PE-Cy7	eBioscience
CD73 *	mouse IgG1	human	AD2	FITC	eBioscience
CD90	mouse IgG1	rat	OX-7	PE-Cy7	BD Biosciences
CD90 *	mouse IgG1	human	5E10	FITC	BD Biosciences
CD105	mouse IgG1	rabbit	SN6	FITC	GeneTex
CD105 *	mouse IgG1	human	266	FITC	BD Biosciences
Vimentin	mouse IgG2a	human	Vim 3B4	-	Dako Cytomation
α-SMA	mouse IgG2a	human	1A4	-	Dako Cytomation
Desmin	mouse IgG1	human	D33	-	Dako Cytomation

* novel antibodies used for the detection of CD73, CD90 and CD105; α-SMA—α smooth muscle actin.

**Table 2:** List of additional primary and secondary antibodies used for confocal microscopy.

Marker	Host	Reactivity	Clone	Conjugate	Company
CD49f	rat IgG1	rabbit	GoH3	-	Biolegend
Sec. Ab	goat	rat	polyclonal	FITC	Biolegend
ALPL	mouse	rabbit	TRA-2-49	-	Novus Biologicals
SOX2	mouse	human	245610	-	R&D Systems
Sec. Ab	goat	mouse IgG	polyclonal	FITC	Bio-Rad
NANOG	goat	human	polyclonal	-	R&D Systems
OCT4	goat	human	polyclonal	-	R&D Systems
Sec. Ab	donkey	goat IgG	polyclonal	FITC	Bio-Rad

Sec. Ab—secondary antibody; ALPL—alkaline phosphatase; SOX2—sex determining region Y—box 2; OCT4—octamer-binding transcription factor 4.

**Table 3:** Nucleotide sequences and size of RT-PCR products.

Gene	Product Size (bp)	Forward Primer	Reverse Primer	Reference
CD29	287	5′-AGAATGTCACCAACCGTAGCA-3′	5′-CACAAAGGAGCCAAACCCA-3′	[35]
CD44	112	5′-TCATCCTGGCATCCCTCTTG-3′	5′-CCGTTGCCATTGTTGATCAC-3′	[11]
CD73	170	5′-CTCCTTTCCTCTCAAATCCAG-3′	5′-GTCCACGCCCTTCACTTTC-3′	[35]
CD90	293	5′-CTGCTGCTGCTCTCACTGTC-3′	5′-ACAGAAGCAGCTTTGGGAAA-3′	[31]
CD105	109	5′-TGACATACAGCACCAGCCAG-3′	5′-AGCTCTGACACCTCGTTTGG-3′	[11]
B2M	118	5′-ATTCACGCCCAATGATAAGG-3′	5′-ATCCTCAGACCTCCATGCTG-3′	[31]
CD34	206	5′-TTTCCTCATGAACCGTCGCA-3′	5′-CGTGTTGTCTTGCGGAATGG-3′	[31]
CD45	262	5′-TACTCTGCCTCCCGTTG-3′	5′-GCTGAGTGTCTGCGTGTC-3′	[35]
ST3GAL2 (SSEA-4)	126	5′-CTGGGAGAATAACCGGTACG-3′	5′-GCTCAGTTGCCTCGGTAGAC-3′	[33]
ALPL (MSCA-1)	137	5′-CCCTCATGTGATGGCTTACG-3′	5′-CTCAGAACAGGACGCTCAGG-3′	[33]
NANOG	122	5′-GCCAGTCGTGGAGTAACCAT-3′	5′-CTGCATGGAGGACTGTAGCA-3′	[31]
OCT4	149	5′-GAGGAGTCCCAGGACATGAA-3′	5′-GTGGTTTGGCTGAACACCTT-3′	[31]
SOX2	152	5′-CAGCTCGCAGACCTACATGA-3′	5′-TGGAGTGGGAGGAAGAGGTA-3′	[31]
ALDH	135	5′-CTGGGAAAAGCAACCTGAAG-3′	5′-AACACTGGCCCTGATGGTAG-3′	NM_001082013.1 ^1^

^1^ NCBI Reference Sequence; B2M—β-2 microglobulin; ST3GAL2—ST3 β-galactoside α-2,3-sialytransferase 2; SSEA-4—stage-specific embryonic antigen 4; ALPL—alkaline phosphatase; MSCA-1—mesenchymal stromal cell antigen-1; SOX2—sex determining region Y—box 2; OCT4—octamer-binding transcription factor 4; ALDH—aldehyde dehydrogenase.

**Table 4:** Nucleotide sequences and size of RT-qPCR products.

	Gene	Product Size (bp)	Forward Primer	Reverse Primer	Reference
rMSCs	ENO2	128	5′- ACACACTCAAGGGGGTCATC -3′	5′- GTCGATGGCTTCCTTTACCA -3′	XM_002712914.3 ^1^
	MAP2	161	5′- CTCACCATGTTCCTGGAGGT -3′	5′- GGAGGAGACGTTGCTGAGTC -3′	XM_017343068.1 ^1^
	B2M	118	5′-CTCCTTTCCTCTCAAATCCAG-3′	5′-GTCCACGCCCTTCACTTTC-3′	[31]
hMSCs	hENO2	238	5′- GGAGAACAGTGAAGCCTTGG -3′	5′- GGTCAAATGGGTCCTCAATG -3′	[37]
	hMAP2	97	5′- AGTTCCAGCAGCGTGATG -3′	5′- CATTCTCTCTTCAGCCTTCTC -3′	[37]
	hACT	125	5′- CCTGGCGTCGTCATTAGTG -3′	5′-TCAGTCCTGTCCATAATTAGTCC-3′	[37]

^1^ NCBI Reference Sequence; rMSCs—rabbit mesenchymal stem cells; hMSCs—human mesenchymal stem cells; ENO2—neuron-specific enolase; MAP2—microtubule-associated protein 2; B2M—β-2 microglobulin; hACT—β-actin.

**Table 5:** Detection of the expression of markers using flow cytometry.

	Percentage of Positive Cells %			
	rAT-MSCs	rBM-MSCs	rAF-MSCs	hAT-MSCs
CD29	92.12 ± 6.65	89.50 ± 8.03 [33]	96.0 ± 5.7 [32]	98.98 ± 0.59
CD34	1.42 ± 0.67	0.78 ± 0.44 [33]	0.37 ± 0.2 [32]	0.77
CD44	97.15 ± 1.45	89.08 ± 8.44 [33]	93.7 ± 2.3 [32]	NT
CD45	1.13 ± 0.40	4.52 ± 2.99 [33]	1.65 ± 1.1 [32]	NT
CD49f	98.92 ± 0.86	79.32 ± 12.63	96.68 ± 1.61	77.90 ± 10.18
CD73	4.10 ± 0.02	3.54 ± 1.83 [33]	7.93 ± 5.0 [32]	NT
CD73 *	60.50 ± 7.37	73.75 ± 21.16	27.23 ± 16.54	98.58 ± 0.32
CD90	10.21 ± 0.07	8.74 ± 4.39 [33]	15.6 ± 4.0 [32]	NT
CD90 *	95.97 ± 3.17	98.23 ± 2.09	70.77 ± 16.55	98.97 ± 0.47
CD105	4.60 ± 0.86	2.03 ± 1.73 [33]	0.56 ± 0.4 [32]	NT
CD105 *	38.83 ± 4.30	73.45 ± 10.96	11.50 ± 3.30	94.44 ± 1.93
Vimentin	85.08 ± 11.43	97.84 ± 3.80 [33]	91.9 ± 4.7 [32]	NT
α-SMA	86.26 ± 10.15	98.75 ± 1.19 [33]	89.0 ± 9.0 [32]	NT
Desmin	75.29 ± 17.09	50.12 ± 11.37 [33]	85.1 ± 9.9 [32]	NT
ALDH	74.63 ± 13.61	70.60 ± 21.38	31.13 ± 8.11	75.56 ± 5.32

* novel antibodies used for the detection of CD73, CD90 and CD105; α-SMA—α smooth muscle actin; ALDH—aldehyde dehydrogenase; rAT-MSCs—rabbit adipose tissue-derived mesenchymal stem cells; rBM- MSCs—rabbit bone marrow mesenchymal stem cells; rAF-MSCs—rabbit amniotic fluid mesenchymal stem cells; hAT-MSCs—human adipose tissue-derived mesenchymal stem cells; NT—not tested.

**Table 6:** Expression of surface markers using ddPCR.

	Percentage of Positive Droplets %		
	rAT-MSCs	rBM-MSCs	rAF-MSCs
CD29	90.3 ± 6.7	94.6 ± 5.4	89.1 ± 7.7
CD44	99.5 ± 0.8	89.9 ± 8.7	89.6 ± 12.6
CD45	0.0 ± 0.0	0.1 ± 0.0	0.0 ± 0.0
CD73	60.7 ± 25.5	42.1 ± 15.4	25.7 ± 16.8
CD90	99.9 ± 0.0	47.9 ± 8.6	58.3 ± 7.4
CD105	55.8 ± 22.2	50.4 ± 28.0	16.1 ± 10.5

rAT-MSCs—rabbit adipose tissue-derived mesenchymal stem cells; rBM-MSCs—rabbit bone marrow mesenchymal stem cells; rAF-MSCs—rabbit amniotic fluid mesenchymal stem cells.

**Table 7:** Summary of characteristic features of rabbit and human mesenchymal stem cells from different tissues.

	Adipose Tissue	Bone Marrow	Amniotic Fluid
rMSCs	PDT: approx. 2 days - Phenotype: CD29^+^; CD44^+^; CD73±; CD90±; CD105±; CD34±; CD45- - SOX2±; OCT4±; NANOG± - Differentiation potential: adipo-genic, osteogenic, chondrogenic, neurogenic [17,34,39,58,60,75,76,77,78,79]	- PDT: approx. 5 days - Phenotype: CD29^+^; CD44^+^; CD73^+^; CD90^+^; CD105^+^; CD14^-^; CD34^¬^-; CD45^-^ - SOX2±; OCT4±; NANOG± - Differentiation potential: adipogenic, osteogenic, chondrogenic, neurogenic [31,33,60,80,81]	- PDT: approx. 3 days - Phenotype: CD29^+^; CD44^+^; CD73±; CD90±; CD105±; CD34 ¬-; CD45- - SOX2±; OCT4±; NANOG± - Differentiation potential: adipogenic, osteogenic, chondrogenic, neurogenic, cardiomyocytes [31,32,33,82,83,84,85]
hMSCs	- PDT: approx. 2 days - Phenotype: CD29^+^; CD44^+^; CD73^+^; CD90^+^; CD105^+^; CD14^-^; CD31^-^; CD34^¬^-; CD45^-^ - SOX2±; OCT4±; NANOG± - Differentiation potential: adipo-genic, osteogenic, chondrogenic, neurogenic, cardiomyocytes, endothelial cells [39,47,63,66,67,68,72,86,87]	- PDT: approx. 6 days - Phenotype: CD29^+^; CD44^+^; CD73^+^; CD90^+^; CD105^+^; CD14^-^; CD34^¬^-; CD45^-^ - SOX2±; OCT4±; NANOG± - Differentiation potential: adipogenic, osteogenic, chondrogenic, neurogenic [47,63,72,80,88]	- PDT: approx. 2 days - Phenotype: CD29^+^; CD44^+^; CD73^+^; CD90^+^; CD105^+^; CD14^-^; CD34 ¬-; CD45^-^ - SOX2±; OCT4±; NANOG± - Differentiation potential: adipogenic, osteogenic, chondrogenic, neurogenic, hepatocyte, epithelilal lung lineages, kidney lineage [40,87,89,90,91,92,93,94,95]

rMSCs—rabbit mesenchymal stem cells; hMSCs—human mesenchymal stem cells; PDT- population doubling time; SOX2—sex determining region Y—box 2; OCT4—octamer-binding transcription factor 4; +—positive expression; -—negative expression; ±—expression differs among studies.

## Data Availability

The data presented in this study are available in the article.

## Appendix A

### Differentiation Assays

To evaluate the multipotent character of rabbit adipose tissue-derived mesenchymal stem cells (rAT-MSCs), cells were differentiated into three basic lineages (adipogenic, chondrogenic and osteogenic) using standard induction media. Differentiation into adipogenic, chondrogenic and osteogenic lineages was performed in accordance with the manufacturer’s instructions of commercially available kits (StemPro^®^ Adipogenesis, StemPro^®^ Chondrogenesis, StemPro^®^ Osteogenesis; Thermo Fisher Scientific). Histological staining was used to evaluate the differential potential, as described in our previous studies [32,33]. Positive staining of proteoglycan deposits with Safranin-O confirmed chondrogenesis (Figure A1D). Stained lipid droplets in the cytoplasm using Oil-Red-O confirmed adipogenic differentiation (Figure A1E). Successful osteogenesis was verified by the detection of calcium aggregates (Figure A1F) stained with Alizarin-Red-S. As a control, non-induced cells were exposed to the staining with the appropriate dyes in recommended time intervals (Figure A1A–C).
